# Formation mechanism and prediction model of juvenile delinquency

**DOI:** 10.3389/fpsyg.2023.1087368

**Published:** 2023-05-02

**Authors:** Shuhui Xu, Junwen Yu, Yu Hu

**Affiliations:** Department of Psychology, School of Education, Wenzhou University, Wenzhou, China

**Keywords:** juvenile delinquency, formation mechanism, prediction model, family factors, social relationships, self-consciousness

## Abstract

Exploring the formation mechanism of juvenile delinquency is of great significance to prevent juvenile delinquency. The present study examined relations and interactions among juvenile delinquents’ self-consciousness, family factors, social relationships, belief in a just world, and legal consciousness, and then developed a predictive model to distinguish between juvenile delinquents and non-delinquents. The results showed that family factors have a significant influence on the formation of juvenile delinquents’ self-consciousness and there are notable differences in family environment and self-consciousness between delinquent and non-delinquent adolescents. Due to the complex interactions among juvenile delinquency’s self-consciousness, family factors, social relationships, belief in a just world and legal consciousness, adolescents’ self- consciousness and social relationships can be utilized to predict and classify the groups of delinquent and non-delinquent adolescents effectively. Therefore, the key to preventing juvenile delinquency is to improve their self-consciousness and develop their prosocial relationships.

## 1. Introduction

Juvenile delinquency is a long-term social problem (Ruoyu, 2020), which leads to the stagnation of individual development and the destruction of social order. Therefore, exploring the formation mechanism and a prediction model on juvenile delinquency can help strengthen the prevention education and correct the behavior of juvenile delinquency. According to the mainstream criminology theory, parental supervision education plays an important role in preventing and managing the anti-social behaviors of children (Wallner et al., 2020). Later relevant studies also confirm the correlation between parenting style and problem behaviors of children and adolescents (Hoeve et al., 2009; Walters, 2019; Ruoyu, 2020). Parental knowledge is an important factor of predicting juvenile delinquency (Glenn and Espelage, 2019). However, few researchers have revealed the formation mechanism from normal teenagers to delinquent teenagers. The purpose of this study is to reveal the initial reasons for the formation of juvenile delinquents and the various factors that influence crime, such as individuals, families, society, values, and the interaction between these factors. Finally, the study is expected to present a key factor in the prediction of juvenile delinquency, that is, the prediction model of juvenile delinquency.

### 1.1. Parenting Style, self-consciousness, and juvenile delinquency

Previous studies have shown that perpetrators’ parents tend to be less warm, authoritarian, harsh, and inconsistent (Hoeve et al., 2008; Tapia et al., 2018). Compared with non-criminal juveniles, the parenting style of criminal juveniles is more manifested as punishment and low support, and the relationship between parents is also less harmonious (Amran and Basri, 2020). For men, low parental support and mothers’ disapproval are independent predictors of crime (Anna and Farrington, 2000). Family stress theory suggested that the relationship between bad neighborhood conditions and crime are influenced by parental behavior, such as supervision (Hoffmann, 2015). More research has confirmed that teenagers in areas where crimes are frequent have less parental support and supervision (Simons and Burt, 2011). Parental monitoring predicts juveniles delinquency (Mohammad and Nooraini, 2021). Teenagers who have parenting styles low on demandingness but high on responsiveness or corporal punishment are more likely to commit crimes later in life than those who have harmonious relationships and low on corporal punishment (Simons and Sutton, 2021). In other words, the family can always be the initial factor in formation of crime.

Self-consciousness is considered as a mental representation of the objective self “me” (public dimension), paying attention also to the “I” (private dimension) and other major dimensions of the self, such as self-experience, self-monitoring, and self-control (Nie et al., 2014). Self-control is the core component of self-consciousness. The establishment of self-consciousness assessment tool is based on self-esteem scale (Delvecchio et al., 2014). So far, there is little research on the relationship between self-consciousness and juvenile delinquency, but there is a lot of research on the relationship between self-control, self-esteem, and juvenile delinquency. Gottfredson and Hirschi (1990) theory of general crime held that a low level of self-control is an important cause of criminal behavior. And he added that the development of self-control depends on how parents raised their children. Empirical research has supported the idea that, on the whole, parents who fail to properly monitor their children, to recognize their children’s antisocial behavior, and ignored them will raise children with low self-control (Tehrani and Yamini, 2020). Hirsch believed that parents have enough time to influence their children until their self-control level is stable at around 8 years old. In other words, the parental early parenting style will affect their children’s level of self-control, which will further influence their criminal possibility (Brauer, 2017). Therefore, the self-control of children is formed in the parenting style of parents and gradually formed in the interaction between parents and children (Li et al., 2019). So how do parents’ parenting styles to contribute to their children’s low self-control? A study has revealed that mothers with low levels of self-control are more likely to engage in ineffective parenting than those with high levels of self-control (Nofziger, 2008). Parenting skills are passed on from generation to generation (Lomanowska et al., 2017), resulting in a stable level of self-control between generations. Then it becomes a vicious circle for families with low self-control. Unfortunately, there is a social selective mating trend where individuals with low levels of self-control prefer those also with low levels of self-control in their spouse selection (Boutwell and Beaver, 2010). This also causes the intergenerational transmission of crime. Family factors, such as the intergenerational spread of parenting styles, may be behind this phenomenon (Farrington et al., 2009). In addition to self-control, research has also shown a link between low self-esteem and crime, and there is no gender difference (Donnellan et al., 2005). Hirsch also pointed out that individuals with low self-control tend to be impulsive, egocentric, fun-loving, and lazy, inclining to lead to criminal behavior. That is to say, the trait of low self-control will further affect the individual’s social relations.

### 1.2. Social relations and juvenile delinquency

Studies have shown that rates of juvenile delinquency and violence are high in unfavorable communities (Wolff et al., 2018). Neighborhood structure and social characteristics, which are important predictors of juvenile delinquency, will pass the risk to adolescents through families and peers (Kennedy et al., 2019). It is certain that social relations in the community will have an impact on teenagers. The disadvantages of the community are indirectly related to juvenile delinquency through parental rearing (Steinberg, 2006; Wang et al., 2020). That is to say, social relations will affect juvenile delinquency. Hirsch later tried to explain juvenile delinquency in terms of social relations, and believed that an individual’s attachment to his family, school, and other important social institutions can predict whether he will commit a crime or not (Brown and Jennings, 2014; Pyle et al., 2020). The later research also judged the development of social relations through the relationship between individuals and their families (Ohtaka and Karasawa, 2019), teachers (Mainhard et al., 2018), and peers (Amati et al., 2018). as well as their belief in law and religion (Bouffard and Rice, 2011). Specifically, community inferior and social disorganization are positively related to youths’ association with deviant friends (Ge et al., 2002). Studies have shown that social relationships are the best predictor of criminal behavior (Brown and Jennings, 2014; Copp et al., 2020). Crime, in turn, weakens social bonds and leads to continued crime. Previous actions are linked to future actions through social bonds (Ford, 2005). These studies suggested that social relations can predict the incidence of crime. In turn, then, anything that weakens social relations could help us understand the evolution of criminal behavior.

### 1.3. The social control theory and juvenile delinquency

Hirsch’s social control theory is one of the most cited theories in criminology. In contrast to earlier criminal theories, this theory focuses on what factors inhibit the occurrence of criminal behaviors, rather than why people commit crimes. According to the social control theory, individuals with good social relations are more likely to follow social norms, while those with poor social relations are more likely to deviate from social norms and commit illegal acts. This theory suggests that individuals are socialized with four social bonds: an intimate relationship with parents or significant others, acceptance of mainstream social values, participation in traditional social activities, and holding a just view of the world (Booth et al., 2008). In other words, if the relationship between the individual and the society is benign, the socialization process can be successfully completed and the individual has various abilities to adapt to society (Gecas, 1982). On the other hand, it is possible to be marginalized by society and turn to crime.

The social control theory believes that the transmission of family values to children and the relationship between children and their parents have an important influence on whether children will commit crimes in the future. Attachment to parents and the recognition and internalization of parents’ traditional values can reduce adolescents’ contacts with peers who commit crimes and the occurrence of criminal behaviors (Robert, 2018). As for the impact of family on crime, the current academic circles analyzed the impact of social capital generated by family on individual life trajectory. For example, the inter-generational transfer of resources is contained in the social relations established by families. When social capital in families and communities dries up, crime rate will tend to rise. Therefore, the family is no longer treated as a static system, but a dynamic social capital system that actively guides adolescents away from criminal risks through parents (Wen, 2017). Parents spend time and energy on creating and maintaining emotional bonds, providing prosaically behavioral guidance with their children, which changes the likelihood that adolescents will join criminal behavior or criminal gangs. Teenagers who benefit from the family investment are more likely to maintain strong emotional attachment to their parents, to have pro-social beliefs, and also to do well in school, which can reduce their chances of joining criminal gangs (Liu and Miller, 2020). Increasing an individual’s attachment to society, accepting traditional norms, and trusting social order to be fair, can reduce crime (Charis and Ronald, 2017). Similar to attachment to family, attachment to school can reduce adolescent delinquency (Lee et al., 2018). This is because less attachment to school is more likely to engage in problematic behavior or have more access to troubled adolescents (Watts et al., 2019). The individual’s acquisition of mainstream values and belief in a just world is also gradually formed in the process of interaction with family and peers.

## 2. Hypotheses

The purpose of this study is to explore the formation mechanism of juvenile delinquency from the perspective of adolescents’ self-factors, family factors, adolescents’ relationship with society, and adolescents’ attitude toward mainstream values, and then construct a prediction model of juvenile delinquency. The research quantified the influence of family factors in the way of parenting, quantified the factors of adolescents themselves in the way of self-consciousness, and quantified the relationship between delinquent adolescents and others and the society in the way of alienation. Alienation is a kind of depressed emotional experience caused by the rupture of the normal social relations between individuals and the society, which is rooted in the relationship between individuals and the society and will act on the relationship between individuals and the society. The research quantified the mainstream values of delinquent adolescents in the way of belief in a just world and legal consciousness. Through sorting out and analyzing relevant theories and literature, this study concluded that family factors can reveal the formation mechanism of juvenile delinquency’s self-consciousness, which includes three dimensions of self-control, self-cognition, and self-experience. However, self-consciousness, interpersonal alienation, and social alienation have interactive effects on juvenile delinquents’ belief in a just world and legal consciousness. We specifically tested these research hypotheses:

*Hypothesis 1*: Family factors, namely family rearing style, have a significant predictive effect on the formation of juvenile delinquency’s self-consciousness. Specifically, a positive family rearing style has a positive prediction effect on the formation of juvenile delinquency’s self-consciousness, while negative family rearing style has a negative prediction effect on the formation of juvenile delinquency’s self-consciousness.

*Hypothesis 2*: The self-consciousness of juvenile delinquents can significantly predict their socialization degree, namely, self-consciousness can effectively predict their interpersonal alienation, social alienation, belief in world justice, and legal consciousness.

*Hypothesis 3*: The alienation of juvenile delinquents has a mediating effect between self-consciousness and belief in a just world. There is also a mediating effect between the sense of alienation and legal consciousness.

*Hypothesis 4*: The nine dimensions of self-consciousness and alienation can effectively distinguish delinquent adolescents from ordinary adolescents. In other words, these two factors can be used as the main factors to predict juvenile delinquency model.

## 3. Materials and methods

### 3.1. Data collection and participants

Research data were collected in batches. The first data contained two groups. The juvenile delinquent group was selected randomly from two sections of the Juvenile Criminal Prison in Shandong province. The study finally kept 403 valid samples. The average age of the first crime of the sample was *M* = 15.85, SD = 1.17, and the average age of the investigation was *M* = 19.71, SD = 2.54. Among them, there were 134 juvenile delinquents with primary school education when they were put into prison, accounting for 33.3% of the total number of respondents. There were 228 juvenile delinquents with middle school education, accounting for 56.6% of the total number. There were 41 juvenile delinquents with high school or technical secondary school education, accounting for 10.2% of the total number. The second group was non-delinquent youth, and 270 men were randomly selected from a middle school as the control group, with an average age of M = 18.42 and SD = 0.54. A total of 256 valid data samples were retained after eliminating invalid questionnaires. The first data is used to investigate the relationship between juvenile delinquents and the family of origin and explored influence of juvenile delinquency’ s self-consciousness on their socialization degree.

The second batch of data was from the juvenile reformatory in Shandong province by randomly selecting two prison districts. A total of 300 valid samples were retained after eliminating invalid data. The average age of the retained samples of the first offense was M = 15.89 and SD = 0.93. The average age of sample at the time of participating in the survey was M = 19.27, SD = 2.17. Among them, there were 78 juvenile delinquents who graduated from primary school when they were put into prison, accounting for 26% of the total number of respondents. There were 184 junior middle school graduates, accounting for 61.3% of the total number. There were 38 high school or technical secondary school graduates, accounting for 12.7% of the total number.

The third batch of data contained two groups. One group was juvenile delinquents and the other was non-delinquent juveniles whose age was close to the juvenile delinquents. The group of juvenile delinquents used the data of the second batch of tests. The non-juvenile delinquents were randomly selected from 5 classes in a middle school in Shandong province, and 236 samples were retained after eliminating invalid data. The average age was M = 16.11, SD = 1.15. All three pieces of data are used to build a prediction model of juvenile delinquency.

The investigation of juvenile delinquents and juvenile students were carried out with the permissions and consent of the relevant institutions (the Juvenile Criminal Prison and schools) and themselves. The data of the three batches was collected by paper-pencil test. The juvenile delinquents were tested by taking one group as a unit in the classroom. The experimenter was a professional with psychological assessment qualification in prison. It also had a doctoral student in psychology as an assistant. Two prison policemen maintained and managed the order. The middle school students were tested by taking one class as a unit and the test was assisted by the head teacher and psychology doctoral students.

SPSS 17.0 are used for statistical analysis of data in this study, including multiple linear regression, independent samples *t*-test, stepwise regression, logistic regression analysis and so on.

### 3.2. Procedure

This study was divided into three steps: the first step was to investigate the relationship between juvenile delinquents and the family of origin, which was expected to prove the influence of family of origin on the formation of adolescent self-consciousness. Second, on the basis of the first step, we explored the influence of juvenile delinquency’s self-consciousness on their socialization degree, which was mainly quantified from the aspects of juvenile delinquency’s interpersonal relationship, social recognition, and mainstream values, such as justice belief and legal consciousness. The third step was to build a prediction model of juvenile delinquency based on the previous two steps, expected to provide empirical support for the prevention of juvenile delinquency.

## 4. Measures

### 4.1. Egna minnen av barndoms uppfostran (EMBU)

Egna minnen av barndoms uppfostran was developed by Perris et al. (1986) and revised by Yue et al. (1993) The questionnaire is a self-rating scale that people answer questions based on participants’ recollection of their parents’ parenting style. There are 115 items in the scale, and the parenting style of father subscale has six dimensions, namely emotional warmth, understanding, punishment, severity, over-interference, favoring subject, rejection, denial, and over-protection. The parenting style of mother subscale has five dimensions: emotional warmth and understanding, punishment and severity, over-interference and over-protection, favoring subject, rejection, and denial. The scale adopts four points score, which has a good reliability and validity index. In this study, alpha coefficients were 0.93 for fathers and 0.97 for mothers.

### 4.2. Adolescent self-consciousness questionnaire (ASC)

The scale was compiled by Nie et al. (2014). The ASC includes 67 items scored on a five-point Likert-type scale. It contains three subscales and nine factors. The self-knowledge subscale includes appearance-self, social-self, and morality appraisement. The self-experience subscale comprehends learning-self, sense of anxiety and sense of satisfaction. The self-control subscale comprises self-consciousness, self-control and self-monitoring. The score of each subscale is the sum of the items included. A high score indicates a good development of the factor, and a high total score indicates a good development of individual self-consciousness. The Cronbach’s alpha coefficient of the total scale was 0.923, and the Cronbach’s alpha coefficients of the subscales were between 0.640 and 0.839. ASC’s split-half reliability’s coefficient was 0.901 and each subscale’s split-half reliability’s coefficient was between 0.635 and 0.813. The results of confirmatory factor analysis showed that the CFA fitting indexes of the model were χ2 = 3784.5, df = 2108, χ2/df = 1.79, NFI = 0.92, CFI = 0.92, RMSEA = 0.053, which indicated that the data and the model were well fitted, and the scale had good structural validity. In this study, the alpha coefficient of the juvenile delinquent sample was 0.86, and the alpha coefficient of the juvenile non-delinquent sample was 0.94.

### 4.3. Adolescent students’ alienation scale (ASAS)

The scale of adolescent students’ alienation scale was developed by Jessor and Jessor (1977) and Yang and Wu (2002) alienation scale was used as the validity criterion, and the results showed that the correlation index was above medium, and the correlation coefficient reached a significant level. The ASAS includes 52 items scored on a seven-point Likert-type scale and contains three subscales, namely, social alienation, interpersonal alienation and environmental alienation. The Cronbach’s α coefficient of the total scale in this study was 0.91 for juvenile delinquents and 0.96 for juvenile non-delinquents.

### 4.4. The just-word fallacy scale

The Just-World Fallacy scale for college students, compiled by Du et al. (2007), consists of 19 items scored on a five-point Likert-type scale including three factors: ultimate justice factor, immanent injustice factor and immanent justice factor. The Cronbach’s α coefficient of the total scale was 0.808, and the three factors of the scales’ Cronbach’s α coefficient was 0.783, 0.666, and 0.640, respectively. The fitting indexes of confirmatory factor analysis were: χ2 = 292.661, df = 149, χ2/df = 1.964, GFI = 0.906, RMR = 0.054, RMSEA = 0.058, IFI = 0.863, TLI = 0.839. In this study, the Just-Word Fallacy scales’ Cronbach’s α coefficient was 0.69, and the Cronbach’s alpha coefficients of the three subscales was between 0.610 and 0.820.

### 4.5. Legal consciousness scale

The self-made legal consciousness scale contains 18 items scored on a four-point Likert-type scale, each of which has a factor loading value of more than 0.4, and the content involves the legal cognition, legal evaluation, legal emotion and judgment of legal value. In this study, the scale’ Cronbach’s coefficient was 0.702, the test–retest reliability was 0.716 after 6 months, indicating that the reliability of the scale was good. The fitting indexes of confirmatory factor analysis were: χ2/df = 1.46, RMSEA = 0.04, IFI = 0.90, TLI = 0.839, GFI = 0.92, AGFI = 0.89.

## 5. Results

### 5.1. Predictive regression analysis of parenting style on self-consciousness

From Table 1, paternal emotional warmth and understanding was a significant predictor of self-knowledge, parents’ parenting style accounted for 14.8 percent of self-knowledge. Fathers’ emotional warmth understanding, fathers’ severe punishment and mothers’ emotional warmth were significant predictors of self-experience, among which fathers’ severe punishment was a negative influence. Parenting style accounted for 21.2 percent of self-experience. Maternal denial was a significant predictor of self-control, and was a negative predictor. The explanation rate of parental rearing style for self-control was up to 20.6%. Fathers’ emotional warmth and understanding and mothers’ refusal and deny were significant predictors of self-consciousness, with an explanatory rate of 23.3%.

**TABLE 1:** Summary table of multiple regression analysis of each dimension of juvenile delinquency’s parenting style to each dimension of self- consciousness.

Predictor variable		Equation 1 self-knowledge		Equation 2 self-experience		Equation 3 self-control		Self-consciousness	
		**β**	***t***	**β**	***t***	**β**	***t***	**β**	***t***
Warmth and understanding (F)		0.294	2.798**	0.258	2.553*	0.121	1.193	0.251	2.520*
Severe punishment (F)		0.005	0.05	-0.233	-2.490*	-0.093	-0.997	-0.131	-1.421
Over-interference (F)		-0.103	-0.991	0.027	0.271	0.147	1.461	0.039	0.398
Favorite subjects (F)		-0.152	-1.394	-0.023	-0.216	-0.082	-0.778	-0.104	-0.990
Rejection and denial (F)		0.023	0.208	0.111	1.014	-0.020	-0.189	0.054	0.497
Over-protection		0.002	0.018	-0.027	-0.262	0.032	0.315	-0.001	-0.010
Warmth and understanding (M)		0.099	0.682	0.338	2.414*	0.246	1.745	0.261	1.877
Rejection and denial (M)		-0.239	-1.706	-0.128	-0.902	-0.443	-3.278***	-0.358	-2.550*
Severe punishment (M)		-0.020	-0.149	-0.023	-0.180	0.103	0.801	0.037	0.292
Favorite subjects (M)		0.035	0.286	-0.056	-0.470	-0.070	-0.602	-0.038	-0.323
Over-protection (M)		0.165	1.082	0.085	-	0.026	0.174	0.055	0.371
Summary of regression model	F	3.496***		5.414***		5.207***		6.053***	
	*R* ^2^	0.148		0.212		0.206		0.233	

*p < 0.05, **p < 0.01, ***p < 0.001 (two-tailed test), the letter F in the table stands for father, similarly, the letter stands M for mother.

### 5.2. The differences between juvenile delinquents and non-juvenile delinquents in self-consciousness and parenting style

Comparing the scores of delinquent adolescents on various factors of self-consciousness with those of non-juvenile delinquents, it was found that there were significant differences in all factors except appearance-self, self-consciousness, and self-monitoring. Moreover, the scores of each dimension and total scores of juvenile delinquent’s self-consciousness were significantly lower than those of non-juvenile delinquents. Specific results are reported in Table 2.

**TABLE 2:** Comparison of the factors of self-consciousness between juvenile delinquents and non-delinquent adolescents.

Factor	Juvenile delinquents (*N* = 348, M/SD)	Non-juvenile delinquents (*N* = 256, M/SD)	*t*
Appearance-self	19.22/3.12	19.44/3.43	-0.56
Social-self	24.59/4.77	28.41/4.90	-6.56***
Morality-appraisement	16.91/4.47	18.31/4.14	-2.63**
Learning-self	28.99/5.88	33.05/6.72	-5.54***
Sense of anxiety	13.59/3.23	14.98/4.33	-2.83**
Sense of satisfaction	19.53/3.53	21.35/4.69	-3.39***
Self-consciousness	28.38/5.51	29.03/6.49	-0.95
Self-control	32.50/6.23	32.66/6.61	-0.28
Self-monitoring	29.63/4.82	34.00/6.44	-5.85***
Self-knowledge	60.82/9.23	66.29/9.54	-4.84***
Self-experience	62.09/8.77	69.48/12.42	-5.15***
Self-control	90.54/11.75	95.96/14.43	-3.16**
Total scores	213.60/25.16	232.08/31.93	-5.55***

**p < 0.01, ***p < 0.001.

Independent sample *t*-test was conducted on six dimensions of emotional warmth and understanding, severe punishment, over-interference, favorite subject, rejection, denial and over-protection of paternal parenting style of juvenile delinquents and non-delinquent adolescents. The results showed that the score of non-delinquent adolescents was higher than the score of juvenile delinquents in terms of emotional warmth and understanding dimension, and the difference was significant. In terms of severe punishment, over-interference and denial, the scores of juvenile delinquents were higher than the scores of non-delinquents, and the differences were significant. There were no significant differences in dimensions of favorite subject and over-protection. As shown in the Table 3.

**TABLE 3:** Comparison of different dimensions of family rearing style (father) between juvenile delinquents and non-juvenile delinquents (M ± SD, *n* = 112).

Variable	Emotion warmth, understanding	Severe punishment	Over-interference	Favoring subject	Rejection, denial	Over-protection
Juvenile delinquents	46.83/11.31	25.12/9.14	23.76/5.04	10.68/3.53	12.40/4.79	13.52/3.42
Non-juvenile delinquents	53.68/11.46	19.18/6.98	21.41/5.66	19.18/6.98	10.14/4.03	13.94/3.67
*t*	−3.67***	4.39***	2.76**	1.76	3.31***	−0.77

**p < 0.01, ***p < 0.001.

Independent sample *t*-test was conducted on five dimensions of emotional warmth and understanding, over-protection and over-intervention, denial, severe punishment and favorite subject in the mothers’ parenting style of juvenile delinquents and non-juvenile delinquents, the results showed that the score of non-juvenile delinquents was higher than that of juvenile delinquents in the dimension of emotional warmth and understanding, and the difference was significant. On the severe punishment dimension, the score of juvenile delinquents was higher than non-juvenile delinquent’s score, and the difference was significant. There were no significant differences in dimensions of over-protection, denial and favorite subject. Specific results were shown in Table 4.

**TABLE 4:** Comparison of differences in different dimensions between the family rearing styles of juvenile delinquents and non- juvenile delinquents (mother) (M ± SD, *n* = 112).

Variable	Emotion warmth, understanding	Over-protection over-interference	Rejection, denial	Severe punishment	Favoring subject
Juvenile delinquents	49.12/12.04	39.11/8.30	15.82/5.42	16.13/6.42	10.72/3.49
Non-juvenile delinquents	55.52/11.58	37.73/8.83	14.52/5.46	12.95/4.66	9.19/5.77
*t*	−2.98**	0.96	1.54	3.53**	1.88

**p < 0.01.

### 5.3. Stepwise multiple regression analysis of self–Consciousness, social alienation, belief in a just world, and legal consciousness

According to equation 1 in Table 5, the three dimensions of self–consciousness, only self-experience entered the regression equation model, and the determination coefficient was 0.084, thus, self-experience can negatively explain the 8.4% variation of sense of alienation. According to equation 2, there were three predictive variables entering the regression model. According to the beta value and determination coefficient, the three dimensions can effectively and negatively explain 16.403% variation of belief in a just world. By equation 3, there were four prediction variables entering the regression model, the variables of sense-of meaninglessness and natural alienation had negative influences on legal consciousness, belief in a just world and self-control had positive influences on legal consciousness. According to the determination coefficient, four predictive variables can account for 18.8% of legal consciousness.

**TABLE 5:** The stepwise regression analyses of juvenile delinquencies each dimension of self-consciousness, belief in a just world, alienation, and legal consciousness.

Predictor variables		Equation 1 (alienation)		Equation 2 (belief in a just world)		Equation 3 (legal consciousness)	
		**β**	***t***	**β**	***t***	**β**	***t***
Self-experience		-0.290	-5.019***				
Loneliness				-0.167	-2.184*	-0.174	-2.618**
Sense-of meaninglessness				-0.184	-2.711**		
Self-alienation				-0.151	-2.319*		
Natural alienation						-0.172	-2.797**
Belief in a just world						0.146	2.800*
Self-control						0.146	2.500*
Summary of regression model	F	25.187***		16.403***		15.182***	
	*R* ^2^	0.084		0.156		0.188	

*p < 0.05, **p < 0.01, ***p < 0.001.

**FIGURE 1:**
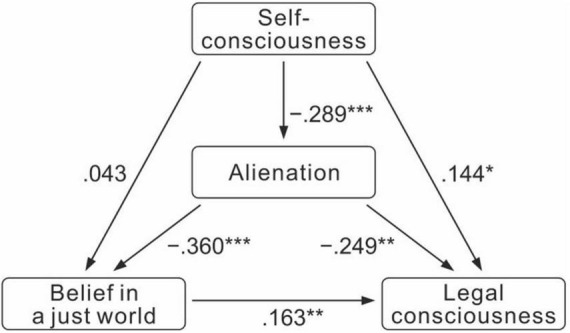
Path analysis diagram of self-consciousness, alienation, belief in a just world and legal consciousness. **p < 0.01 and ***p < 0.001.

In order to focus on the causal relationship between variables, a causal model graph was drawn. It can be seen from Figure 1 that the model graph contains three regression analysis models: the first regression analysis model takes self-consciousness as the independent variable and the total sense of alienation as the dependent variable. The second regression model takes self-consciousness and alienation as independent variables and belief in a just world as dependent variables. The third regression analysis model takes self-consciousness, alienation and belief in a just world as independent variables and legal consciousness as dependent variable. Forced entry method was adopted for analysis, and the results were shown in Table 6.

**TABLE 6:** Summary table of three regression analysis results.

Predictor variable		Equation 1 (alienation)		Equation 2 (belief in a just world)		Equation 3 (legal consciousness)	
		**β**	***t***	**β**	***t***	**β**	***t***
Self-consciousness		−0.289	−5.001***	0.043	0.726	0.144	2.459*
Sense of alienation				−0.360	−6.079***	−0.249	−3.966***
Belief in a just world						0.163	2.681**
Summary of regression model	F	25.013***		21.923***		17.716***	
	*R* ^2^	0.084		0.141		0.158	

*p < 0.05, **p < 0.01, ***p < 0.001.

The path coefficient and related statistics of the above path analysis were filled into the theoretical model figure, as shown in Figure 1.

According to the standardized regression coefficient among variables, the direct effect values of each exogenous variable on the endogenous variable can be obtained: the direct effect values of self-consciousness on belief in a just world, legal consciousness and alienation on the three endogenous variables were 0.043, 0.144, and −0.289, respectively. The direct effect values of alienation on belief in a just world and legal consciousness were −0.360 and −0.249, respectively. The direct effect value of belief in a just world on the endogenous variable of legal consciousness was 0.163. The significance test of five path coefficients in the path analysis model reached the significance level of 0.05.

Indirect effect: the indirect effect value of self-consciousness on the belief in a just world variable was equal to −0.289 × −0.36 = 0.104. The indirect effect value of self-consciousness on the variable of legal consciousness was equal to −0.289 × −0.249 = 0.072. The indirect effect value of alienation on legal consciousness variable was equal to −0.36 × 0.163 = −0.059. According to the basic principle: the direct effect is greater than the indirect effect, indicating that the intermediary variable does not play a role. The direct effect is less than the indirect effect, indicating that the mediation variable has an influence. Therefore, the sense of alienation was not the key factor that self-consciousness affected belief in a just world and legal consciousness. The factor of belief in a just world had a mediating effect on alienation and legal consciousness.

### 5.4. The logistic analysis model of juvenile delinquency and non-juvenile delinquency

As can be seen from Table 7, a total of 12 independent variables were put into the model from the three dimensions of self-consciousness and the nine dimensions of adolescent alienation. The results showed that the independent variables had a significant prediction on juvenile delinquency and juvenile non-delinquency. The significance test of the whole model reached the level of 0.001 (χ2 = 196.407, *p* = 0.000 < 0.001). Hosmer-Lemeshow test value was 6.636 (*p* > 0.05), which did not reach a significant level, indicating that the fitness of the regression model established by 12 independent variables of self-consciousness and alienation was very ideal. In terms of correlation strength coefficient, Cox-Snell *R*^2^ = 0.327, Nagelkerke *R*^2^ = 0.438, indicating a moderately strong correlation between independent variables and dependent variables. Twelve independent variables can explain 32.7, 43.8% of the total variation of juvenile delinquency variables.

**TABLE 7:** Summary on the fitness test of the whole model and the significance test of individual parameters.

The variable name	β	S.E.	Wals	df	Correlation intension
Self-knowledge	−0.119	0.108	1.231	1	Cox-Snell *R*^2^ = 0.327
Self-experience	−0.793	0.125	40.207***	1	Nagelkerke *R*^2^ = 0.438
Self-control	0.217	0.119	3.318	1	
Sense of meaninglessness	0.023	0.185	0.016	1	
Self-alienation	−0.723	0.143	25.448***	1	
Loneliness	−0.529	0.178	8.867**	1	
Natural alienation	0.363	0.132	7.597**	1	
Family alienation	0.030	0.105	0.079	1	
Alienation from living environment	−0.148	0.116	2.433	1	
Social isolation	0.314	0.167	3.543	1	
Oppression and alienation	−0.070	0.132	0.284	1	
Sense of uncontrollability	0.148	0.144	1.061	1	
Constant	9.223	1.402	43.265	1	
Overall model fitness test	Chi-square = 196.407*** Hosmer-Lemeshow test-value = 6.636 n.s.				

**p < 0.001, ***p < 0.001, n.s. p > 0.05.

According to the significance indexes of individual parameters, the Wals values of self-experience, self-alienation, loneliness and natural alienation were 40.207, 25.448, 8.867, and 7.597, respectively, all reaching the significant level of 0.05. The results showed that these four independent variables were significantly correlated with whether the juvenile belonged to a criminal group or not, and these four variables can effectively predict and explain the groups of juvenile delinquency and non-delinquency.

According to the cross table of prediction classification accuracy in Table 8, the original observed values of 220 non-criminal juveniles were classified and predicted according to the logistic regression model. A total of 146 juveniles were classified as non- juvenile delinquents s (correctly classified) and 74 juveniles were classified as juvenile delinquents (incorrectly classified). The original observations of 275 juvenile delinquents were classified and predicted by logistic regression model. A total of 233 juvenile delinquents were classified correctly and 42 juvenile delinquents were classified incorrectly. The percentage of the overall classification is (146 + 233) ÷ 495 = 76.5%.

**TABLE 8:** Crosstab of predictive classification accuracy.

		Predictive value		Correct percentage
		**Non-juvenile delinquents**	**Juvenile delinquents**	
Actual value	Non-juvenile delinquents	146	74	66.4
	Juvenile delinquents	42	233	84.7
Total prediction accuracy				76.5

## 6. Discussion

In parenting style, fathers’ emotional warmth and understanding can significantly positively predict juvenile delinquents’ self-cognition, with an explanation rate of 14.8. In other words, fathers’ emotional warmth and understanding are key factors in the formation of juvenile delinquents’ self-cognition. The reason may be that the samples of juvenile delinquents in this study are all men, so they have a higher identification of fathers. Both fathers’ and mothers’ emotional warmth can significantly positively predict juvenile delinquents’ self-experience, while fathers’ severe punishment significantly negatively predicts juvenile delinquents’ self-experience. Self-experience belongs to emotional experience, so parental emotional support has a significant positive impact on it, while fathers’ severe punishment has a negative impact, which can be explained by gender difference. As for self-control, only mother’s denial has a significantly negative predictive effect on juvenile delinquency. As mentioned in the previous literature review, self-control is formed in early childhood, and the status of mothers is very important in the early parent-child relationship. No matter the time spent together or the actual situation, mother is the main nurturer, so the rejection of mothers can significantly predict self-control. Overall, the emotional warmth and understanding of fathers and the denial of mothers significantly predict the level of self-consciousness of juvenile delinquents. From the side, it reflects the juvenile delinquents’ desire for emotional support, acceptance, and understanding from their parents.

Independent sample *t*-test was performed in the two groups, the results showed that most dimensions of self-consciousness of juvenile delinquents’ scores are significantly lower than non-juvenile delinquents’ scores. To some extent, this result can reveal the self-factors of juvenile delinquency, namely, the low self-consciousness is a key factor of juvenile delinquency. Further comparing the parenting styles of the two groups, the results showed that the parenting styles of juvenile delinquents are less emotional support, severer, and more denial. The above results showed that parenting styles can significantly predict the level of juvenile delinquents’ self-consciousness. The significant difference in self-consciousness and parenting style between the two groups further verified the influence of family factors on juvenile delinquency. Hypothesis one has been proved.

This study evaluated the relationship between juvenile delinquents and society by measuring their sense of alienation, and evaluated their mainstream values by measuring their belief in a just world and legal consciousness. Results showed that the self-experience has a significantly negative effect on sense of alienation. The dimensions of loneliness, meaninglessness, and self-alienation in the sense of alienation have significantly negative predictive effects on the belief in the just world. The self-control, the belief in a just world, and the several dimensions of alienation have significantly predictive effects on the legal consciousness of juvenile delinquency. In other words, based on research one, it is concluded that the self-experience of juvenile delinquents has a predictive effect on their interpersonal and social relations, and meanwhile self-consciousness and alienation have a predictive effect on their belief in a just world. Finally, the three jointly have a predictive effect on their legal consciousness. The path analysis diagram shows the interaction among self-consciousness, alienation, belief in a just world, and legal consciousness. Thus, hypothesis two and hypothesis three have been proved. In short, under the comprehensive effect of family, social and cultural values, the juveniles have low legal consciousness and become criminals for breaking the criminal law. In the whole process, family influence plays a fundamental role. Due to bad parenting styles, their self-consciousness level is low, which affects their relationship with the society. In the process of interaction with others and the society, they do not recognize and accept the mainstream values, and finally break the law that represents the mainstream values culture and become juvenile delinquents.

In the end, based on the previous studies, a prediction model is established to distinguish delinquent adolescents from non-delinquent ones by using three dimensions of self- consciousness and nine dimensions of alienation. The classification accuracy of the model is up to 76.5%, indicating that the model has a strong ability to distinguish. This further proves that self-consciousness and alienation can be used as key self-factors and social factors to predict juvenile delinquency. By analyzing the factors influencing the formation of self-consciousness and alienation, we can reveal the internal mechanism of the formation of juvenile delinquency.

## 7. Limitations

When interpreting the results of this study, there are several limitations. First, the data of each variable is based on the self-report of juvenile delinquents and non-delinquents, which affects the objectivity of the data to some extent. Thus, to solve this problem, other data sources, such as collecting relevant data from teenagers’ parents and friends, can be used later. Data can also be collected from the observations of neighborhood or community. The objectivity and accuracy of data can be confirmed by promoting the diversity of data sources and then verifying them with each other as a whole. Second, the overall sample size of women juvenile delinquents in China is small and they are held in women’s prisons, so the samples of juvenile delinquents in this study are all men. It is still unknown whether the exploration of the formation mechanism and the prediction model of juvenile delinquency finally established are suitable for women juvenile delinquency, that is, whether there are gender differences. Next, women samples can be introduced to detect gender differences. Third, the data in this study is still cross-sectional, so time series tests cannot be conducted. For example, the current measurement of adolescent parenting styles, based on the theory that parenting styles are relatively stable or the change of parenting styles will lead to changes in the corresponding relationship (Patterson et al., 2000). But in fact, our hypothesis based on the influence of family on adolescent self-consciousness may also be the result of other social relations. Therefore, it is still necessary to collect longitudinal data in future research.

## 8. Conclusion

In summary, this study integrated individual, family, and socio-cultural factors to explain the formation mechanism of juvenile delinquency and verified the previous criminological theories. The results confirmed that the root of juvenile delinquency lies in the personality defects caused by poor family education, namely the low self-consciousness. The low self-consciousness continues to affect teenagers’ interpersonal and social relations, which indirectly leads to teenagers’ rejection of mainstream cultural values, and then results in the formation of criminal personality. Finally, based on the previous research, this study proposed a structural model to predict juvenile delinquency, which can be used to judge juvenile delinquency tendency through the level of self-consciousness, interpersonal alienation, and social alienation. This has important educational significance to the prevention of juvenile delinquency. In other words, helping individuals form a high level of self-consciousness and establish a good interpersonal relationship, can effectively prevent juvenile delinquency.

## Data availability statement

The raw data supporting the conclusions of this article will be made available by the authors, without undue reservation.

## Ethics statement

The studies involving human participants were reviewed and approved by the Research Center of Psychology and Behavior. Written informed consent to participate in this study was provided by the participants’ legal guardian/next of kin. Written informed consent was obtained from the individual(s) and minor(s)’ legal guardian/next of kin, for the publication of any potentially identifiable images or data included in this article.

## Author contributions

SX, YH, and JY conceived the study successfully. SX collected the data. SX and JY contributed to data analysis and prepared the manuscript. All authors contributed to the article and approved the submitted version.
